# Domestic Cat Hepadnavirus and Lymphoma

**DOI:** 10.3390/v15122294

**Published:** 2023-11-23

**Authors:** Julia A. Beatty, Thomas Tu, Patricia A. Pesavento, Joao P. Cavasin, Min-Chun Chen, Jonathan A. Lidbury, Joerg M. Steiner, Vanessa R. Barrs, John M. Cullen

**Affiliations:** 1 Department of Veterinary Clinical Sciences, Jockey Club College of Veterinary and Life Sciences, City University of Hong Kong, Hong Kong SAR, China; vanessa.barrs@cityu.edu.hk; 2 Centre for Animal Health and Welfare, City University of Hong Kong, Hong Kong SAR, China; 3 Storr Liver Centre, Westmead Clinical School and Westmead Institute for Medical Research, Faculty of Medicine and Health, The University of Sydney, Westmead, NSW 2145, Australia; t.tu@sydney.edu.au; 4 Sydney Infectious Diseases Institute, University of Sydney at Westmead Hospital, Westmead, NSW 2145, Australia; 5 Department of Pathology, Microbiology & Immunology, School of Veterinary Medicine, University of California, 4206 Vet Med 3A, Davis, CA 95616, USA; papesavento@ucdavis.edu; 6 Gastrointestinal Laboratory, College of Veterinary Medicine and Biomedical Sciences, Texas A&M University, College Station, TX 77843, USA; jcavasin@cvm.tamu.edu (J.P.C.); jlidbury@cvm.tamu.edu (J.A.L.); jsteiner@cvm.tamu.edu (J.M.S.); john_cullen@ncsu.edu (J.M.C.); 7 College of Veterinary Medicine, North Carolina State University, Raleigh, NC 27606, USA

We write to comment on Piewbang C et al. [1].

Whether or not infection of cats with domestic cat hepadnavirus (DCH), a relative of human hepatitis B virus (HBV), may be associated with an increased risk of lymphoma in cats is an interesting question. Several epidemiological studies have reported an increased risk of non-Hodgkin’s lymphoma in HBV-infected humans [2], but we are not aware that any similar risk has been implied or identified in chronic hepadnavirus infection in other species (e.g., ducks, woodchucks, chimpanzees, or ground squirrels).

We believe it is important to highlight that the study design used by Piewbang et al. [1], a comparison of the frequency of DCH DNA detection in blood and lymphoid tissue of cats with and without a diagnosis of lymphoma, does not address the existence of an association between DCH infection and feline lymphoma.

Firstly, the approach used by Piewbang et al. [1] cannot identify cats with undetectable serum DCH DNA that remain persistently infected. Using an assay for DCH anti-core antibody, Fruci et al. [3] showed that more than twice as many cats tested positive for DCH anti-core antibody than were positive for serum DCH DNA. We agree with the authors’ comments in their discussion suggesting that the immune dysfunction associated with B-cell lymphoma, its treatment, and/or concurrent retrovirus infection could increase circulating DCH levels, and therefore may bias the results towards DCH DNA detection in these cats. 

Secondly, Piewbang et al. [1] conducted immunohistochemistry and in situ hybridization (ISH) of lymphoma tissues from cats that tested positive for serum DCH DNA. We believe that the results as presented are inconclusive; the images are difficult to discern due to their non-specific background staining and a comparatively weak signal in the lymphocyte population. The ISH data (reportedly from a single B cell lymphoma) do not include negative controls using an unrelated probe on serial sections at the same magnification (the negative control shows a different field at a different magnification), nor were additional negative controls (for example, non-lymphoma tissues) probed for DCH. The last point is particularly important given that the cytoplasmic, pink, non-punctate staining observed is also the common background pattern of some epithelial cells (e.g., thymic epithelium, hepatocytes), lipid rich-tissues (adrenal cortex, adipose tissue, renal tubular cells), and tissue-bound histiocytes because of their endogenous alkaline phosphatase activity. Thus, any implication that these data support a direct relationship between DCH infection and feline lymphoma, or even lymphotropism, would be misleading.
